# Cognitive deficits associated with novel intrathecal anti-nuclear antibodies

**DOI:** 10.1038/s41380-024-02435-6

**Published:** 2024-02-02

**Authors:** Alexander Maier, Kathrin Nickel, Katharina Domschke, Nils Venhoff, Ludger Tebartz van Elst, Harald Prüss, Dominique Endres

**Affiliations:** 1 Department of Psychiatry and Psychotherapy, Medical Center - University of Freiburg, Faculty of Medicine, University of Freiburg, Freiburg, Germany; 2 Department of Rheumatology and Clinical Immunology, Medical Center - University of Freiburg, Faculty of Medicine, University of Freiburg, Freiburg, Germany; 3 Department of Neurology and Experimental Neurology, Charité - Universitätsmedizin Berlin, Berlin, Germany; 4 German Center for Neurodegenerative Diseases (DZNE) Berlin, Berlin, Germany

**Keywords:** Psychiatric disorders, Neuroscience, Diagnostic markers

## Introduction

Cognitive deficits and neurodegenerative disorders can be associated with inflammation and immune activation detectable by analyzing the cerebrospinal fluid (CSF) [1–6]. Here, a paradigmatic case of a patient with a long-lasting neurocognitive syndrome without evidence of a well-known neurodegenerative disorder but with suspected novel autoantibodies in the CSF is presented.

## Case Study

**Fig. 1:**
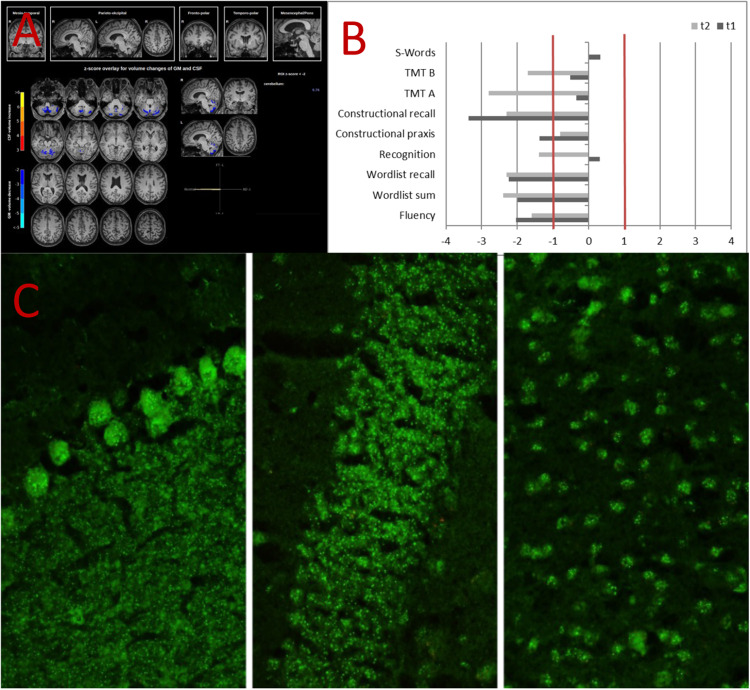
Diagnostic findings. **A** Automated magnetic resonance imaging analysis (https://www.veobrain.com/?page=veomorph) detected slight cerebellar volume loss. **B** The neuropsychological test battery of the Consortium to Establish a Registry for Alzheimer’s Disease (CERAD) in 2021 showed clear evidence of below average performance in verbal learning and retrieval, visual–spatial memory, and cognitive speed; semantic and verbal fluidity were also below average. The CERAD neuropsychological test battery results were relatively similar in 2022. Due to the chronified course of the disease, cognitive improvement under immunotherapy seemed rather unlikely. Therefore, after a multidisciplinary case discussion and consideration of the patient’s preferences, no immunotherapy was tried. **C** Tissue-based assays using cerebrospinal fluid (CSF) material on unfixed murine brain slices [8, 9] showed very strong IgG binding to cell nuclei at multiple spots. All tests in CSF and/or serum for well-characterized immunoglobin (Ig) G autoantibodies against neuronal intracellular (Yo, Hu, Ri, CV2, CRMP5, Ri, Ma1, Ma2, SOX1, Tr(DNER), Zic4, GAD65 amphiphysin), neuronal cell-surface (NMDA-R, LGI1, CASPR2, GABA-B-R, AMPA1-R, AMPA2-R, DPPX), and glial (AQP4, MOG) antigens (not shown here) were negative [cf. 10]. Therefore, a novel autoantibody targeting a nuclear antigen was suspected.

A 57-year-old female patient, who gave written informed consent for publication of this case study, presented in 2022 with a chronic neurocognitive syndrome persisting for over 10 years. She reported having problems with memory, especially remembering names and numbers (e.g., for her banking account) and reduced concentration as well as a deceleration in her performance of housework tasks since approximately 2008. Previously, the patient was mentally healthy, and there was no intellectual disability. Over the last few years, her cognitive deficits showed only a slow deterioration. There was a comorbid migraine without aura. An external MRI performed in 2015 showed no classical signs of a neurodegenerative or inflammatory disorder and only a small expansion of the outer parietal CSF spaces. Cognitive testing in 2021 using the neuropsychological test battery of the Consortium to Establish a Registry for Alzheimer’s Disease (CERAD) showed clear evidence of below average performance with a mild cognitive impairment. A subsequent MRI in 2021 showed no relevant changes. Results of [^18^F]fluorodeoxyglucose (FDG) positron emission tomography (PET) and Tau-PET were normal. Approximately one year later (in 2022), the patient was admitted to our day clinic due to additional intermediate-grade depressive symptoms and for diagnostic work-up [7]. At admittance, the patient was taking topiramate and venlafaxine. Topiramate was discontinued due to possible negative effects on cognition. Depressive symptoms were regredient under multimodal therapy, while neurocognitive symptoms persisted. The CERAD neuropsychological test battery results were mostly unchanged in 2022, as were the results of a follow-up MRI (Fig. 1A, B). Automated MRI analyses (https://www.veobrain.com/?page=veomorph) detected slight cerebellar volume loss. The patient’s history did not show evidence of alcohol abuse. Blood analysis of established rheumatological markers (including indirect immunofluorescence [IIF] on human epithelial type 2 [HEp-2] cells, the routine technology for ANA detection, and testing for extractable nuclear antigens [ENAs]) yielded unremarkable findings. All CSF dementia markers (tau, p-tau, ß-amyloid-quotient) were normal. However, the white blood cell (WBC) count in CSF was borderline elevated, with 5 cells/μL (reference < 5 cells/μL). Furthermore, in IIF on unfixed mouse brain sections [8, 9], a very strong IgG binding to cell nuclei (multiple spots) in CSF was identified (Fig. 1C). The serum showed much weaker autoantibody binding indicating intrathecal synthesis. The patient had no systemic signs of a mixed connective tissue disorder. All tests in serum and CSF for well-characterized immunoglobin (Ig) G neuronal and glial autoantibodies were negative [cf. 10].

## Discussion

This case study describes a patient with a chronic neurocognitive syndrome without relevant signs of a neurodegenerative cause but with signs of neuroinflammation. The clinical course (no relevant deterioration discovered in neuropsychological follow-up), non-specific MRI changes, normal PET results, and negative CSF dementia markers suggest that this is not a case of neurodegenerative dementia. Indications for a possible autoimmune process include the CSF findings of borderline elevated WBC counts and CSF-dominant strong IgG-bindings against cell nuclei with multiple spots on unfixed mouse brain representing potentially novel neuronal autoantibodies. The diagnostic criteria for possible autoimmune encephalitis [11] were not fulfilled due to the time course (a subacute onset within 3 months is required). The additional clinical and diagnostic criteria were also not fulfilled, whereby the CSF WBC count was borderline. The same was true for the criteria of possible autoimmune psychosis [12]. Here, the criteria for clinical symptomatology and the temporal course (as there was no psychosis with a subacute onset) were not fulfilled. Therefore, this case shows the complexity of dealing with slowly progressive neurocognitive syndromes with only borderline pathological diagnostic findings. The link between cognitive deficits and neuroinflammation is well known from Alzheimer’s disease (AD) research. In the mouse model of AD, behavioral deficits can develop in parallel with neuroimmunological processes and may precede the neuropathology typical of AD [13].

Three scenarios are possible regarding the pathophysiological role of anti-nuclear autoantibodies in this constellation:

1. Autoantibodies do not play a role. Various anti-nuclear autoantibodies are found in the serum of patients lacking any clinical symptoms of autoimmune disorder [14]. They may then just be there as part of an unknown immunological process or even as natural autoantibodies in susceptible individuals. In addition, autoantibodies targeting intracellular antigens (such as nuclear epitopes) were traditionally believed to not being clinically relevant, as they can hardly reach their target in vivo. In the presented case, however, there was evidence of intrathecal autoantibody synthesis, and the autoantibodies were binding specifically to brain cells.
2. Autoantibodies play a modulatory role in the context of a process (similar to paraneoplastic or virus-induced autoantibody production) that is not yet understood. This is already assumed for other well-characterized neuronal autoantibodies (e.g., IgA or IgM anti-NMDA-R autoantibodies) [4, 5].
3. Autoantibodies are causally responsible for the neurocognitive syndrome. A novel nuclear antigen could be responsible (since the testing for well-characterized anti-nuclear autoantibodies on HEp-2 cells and for all known ENAs remained negative). Even though potentially only small amounts of the autoantibodies reach their intracellular antigen, the high CSF-dominant autoantibody titer and the long exposure time of likely several years may be sufficient to cause subtly, slowly progressing neuronal dysfunctions leading to the here observed chronic neurocognitive syndrome.

The last two scenarios would have potential therapeutic consequences. Clinical confirmation of causality is difficult as successful autoantibody removal may require long treatment periods before a beneficial effect can be observed. To prove scenarios 2 and 3 experimentally, the autoantigens of such rare autoantibody patterns should be characterized, followed by investigation of the functional consequences of the autoantibodies in neuronal cultures and animal models [cf. 2]. Results emerging from this research pipeline will likely guide clinical decisions and offer immunotherapeutic possibilities for a subgroup of similar patients in the future [cf. 8].

## Supplementary information

Supplemental Table 1

## Data Availability

All necessary data can be found in the paper.

## References

[1] Gigase FAJ, Smith E, Collins B, Moore K, Snijders GJLJ, Katz D, et al. The association between inflammatory markers in blood and cerebrospinal fluid: a systematic review and meta-analysis. Mol Psychiatry. 2023;28:1502–15. 10.1038/s41380-023-01976-6. 37055513 10.1038/s41380-023-01976-6 PMC10266485

[2] Prüss H. Autoantibodies in neurological disease. Nat Rev Immunol. 2021;21:798–813. 10.1038/s41577-021-00543-w. 33976421 10.1038/s41577-021-00543-w PMC8111372

[3] Snyder A, Grant H, Chou A, Lindbergh CA, Kramer JH, Miller BL, et al. Immune cell counts in cerebrospinal fluid predict cognitive function in aging and neurodegenerative disease. Alzheimers Dement. 2023. 10.1002/alz.12956. 10.1002/alz.12956 PMC10425564 36791265

[4] Bartels F, Wandrey MM, Aigner A, Strönisch T, Farmer K, Rentzsch K, et al. Association Between Neuronal Autoantibodies and Cognitive Impairment in Patients With Lung Cancer. JAMA Oncol. 2021;7:1302–10. 10.1001/jamaoncol.2021.2049. 34196651 10.1001/jamaoncol.2021.2049 PMC8251651

[5] Doss S, Wandinger KP, Hyman BT, Panzer JA, Synofzik M, Dickerson B, et al. High prevalence of NMDA receptor IgA/IgM antibodies in different dementia types. Ann Clin Transl Neurol. 2014;1:822–32. 10.1002/acn3.120. 25493273 10.1002/acn3.120 PMC4241809

[6] Pisetsky DS, Lipsky PE. New insights into the role of antinuclear antibodies in systemic lupus erythematosus. Nat Rev Rheumatol. 2020;16:565–79. 10.1038/s41584-020-0480-7. 32884126 10.1038/s41584-020-0480-7 PMC8456518

[7] Runge K, Reisert M, Feige B, Nickel K, Urbach H, Venhoff N, et al. Deep clinical phenotyping of patients with obsessive-compulsive disorder: an approach towards detection of organic causes and first results. Transl Psychiatry. 2023;13:83. 10.1038/s41398-023-02368-8. 36882422 10.1038/s41398-023-02368-8 PMC9992508

[8] Kreye J, Reincke SM, Kornau HC, Sánchez-Sendin E, Corman VM, Liu H, et al. Therapeutic Non-self-reactive SARS-CoV-2 Antibody Protects from Lung Pathology in a COVID-19 Hamster Model. Cell. 2020;183:1058–e19. 10.1016/j.cell.2020.09.049. 33058755 10.1016/j.cell.2020.09.049 PMC7510528

[9] Franke C, Boesl F, Goereci Y, Gerhard A, Schweitzer F, Schroeder M, et al. Association of cerebrospinal fluid brain-binding autoantibodies with cognitive impairment in post-COVID-19 syndrome. Brain Behav Immun. 2023;109:139–43. 10.1016/j.bbi.2023.01.006. 36657623 10.1016/j.bbi.2023.01.006 PMC9841734

[10] Endres D, von Zedtwitz K, Matteit I, Bünger I, Foverskov-Rasmussen H, Runge K, et al. Spectrum of Novel Anti-Central Nervous System Autoantibodies in the Cerebrospinal Fluid of 119 Patients With Schizophreniform and Affective Disorders. Biol Psychiatry 2022;92:261–74. 10.1016/j.biopsych.2022.02.010. 35606187 10.1016/j.biopsych.2022.02.010

[11] Graus F, Titulaer MJ, Balu R, Benseler S, Bien CG, Cellucci T, et al. A clinical approach to diagnosis of autoimmune encephalitis. Lancet Neurol. 2016;15:391–404. 10.1016/S1474-4422(15)00401-9. 26906964 10.1016/S1474-4422(15)00401-9 PMC5066574

[12] Pollak TA, Lennox BR, Müller S, Benros ME, Prüss H, Tebartz van Elst L, et al. Autoimmune psychosis: an international consensus on an approach to the diagnosis and management of psychosis of suspected autoimmune origin. Lancet Psychiatry. 2020;7:93–108. 10.1016/S2215-0366(19)30290-1. 31669058 10.1016/S2215-0366(19)30290-1

[13] Marchese M, Cowan D, Head E, Ma D, Karimi K, Ashthorpe V, et al. Autoimmune manifestations in the 3xTg-AD model of Alzheimer’s disease. J Alzheimers Dis. 2014;39:191–210. 10.3233/JAD-131490. 24150111 10.3233/JAD-131490 PMC4376359

[14] Pisetsky DS. Antinuclear antibody testing - misunderstood or misbegotten? Nat Rev Rheumatol. 2017;13:495–502. 10.1038/nrrheum.2017.74. 28541299 10.1038/nrrheum.2017.74

