# Supplementary material for: Cognitive deficits associated with novel intrathecal anti-nuclear antibodies
Source: Mol Psychiatry. 2024 Feb 2;29(6):1906–8. doi: 10.1038/s41380-024-02435-6 (PMC11371633; doi:10.1038/s41380-024-02435-6)
Supplement: Supplementary file 1 — Supplemental Table 1 [file 41380_2024_2435_MOESM1_ESM.pdf]

SUPPLEMENTAL TABLE

**Serum antibodies, immunological markers and serologies**	
Anti-thyroid antibodies (against TPO, TG and TSH-receptor)	Negative
ANAs (*on HEp-2 cells*), ANCAs (*on EthOH-/formalin-fixed neutrophils*), APAs	ANAs, APAs and ANCAs negative
ENA antibodies (*Anti-snRNP/Sm, Anti-Sm, Anti-SS-A/Ro, Anti-Ro-52, Anti-SS-B/La, Anti-Scl-70, Anti-PM-Scl, Anti-Jo1, Anti-centromer, Anti-PCNA, Anti-nucleosomes, Anti-histone, Anti-ribosomal p-protein, Anti-AMA-M2, Anti-DFS70*)	Negative
Complement factors (C3, C4)	Normal
IgG, IgM, and IgA levels	Normal
CRP	4.5 mg/l (ref.: < 5 mg/l)
Anti-streptolysin-O	Normal
Anti-DNaseB	Normal
Serology for Lyme disease or lues	Negative
Paraneoplastic IgG antibodies against intracellular antigens	Negative
Well-characterized neuronal IgG cell surface antibodies	Negative
Anti-MOG/AQP4-IgG antibodies	Negative
Tissue based assay on unfixed murine brain tissue (Prof. Prüss, Charité Berlin)	**Moderate IgG binding against cell nuclei**
Serologies (CMV, EBV, HBV, HCV, HIV, tuberculosis)	Negative
**Cerebrospinal fluid**	
White blood cell count	**5/µL** (ref.: <5/µL)
Protein concentration	246 mg/L (ref.: <450 mg/L)
Albumin quotient	3.8 (ref.: <6.5)
IgG-index	0.44 (ref.: <0.7)
Oligoclonal bands in serum/CSF	Negative
Well-characterized neuronal IgG cell surface antibodies	Negative
MRZ Reaction	Negative
Tissue based assay on unfixed murine brain tissue (Prof. Prüss, Charité Berlin)	**Very strong IgG binding to cell nuclei with multiple spots**

**MRI of the neurocranium**<br><br>Visual inspection<br>Automated morphometry	 No typical atrophies, **borderline expansion of the outer CSF spaces**. Not progredient since a prior MRI in 2015.<br>**Cerebellar volume reduction.**
**EEG**<br>Visual analyses	 No intermittent/ generalized slowing, no epileptic activity
**FDG-PET of the brain** (2021)<br>**TAU-PET of the brain** (2021)	Normal<br>Normal
**OCT**<br>**ERG**<br>a-wave, b-wave, photopic negative response	Normal<br><br>Normal
**Neuropsychological tests (CERAD in 09/2022 and 09/2021)**	**Mild Cognitive Impairment in 2022**, no relevant progression compared to prior testing in 2021

Supplemental Table 1: Full diagnostic findings during admission 2022. ↑ means increased. *Abbreviations: ANAs, antinuclear antibodies; ANCAs, anti-neutrophil cytoplasmic antibodies; MOG, myelin oligodendrocyte glycoprotein; APAs, antiphospholipid antibodies; AQP4, aquaporin-4; DNase, deoxyribonucleic B; CMV, cytomegalovirus; CERAD, Consortium to Establish a Registry for Alzheimer's Disease; CRP, C-reactive protein; CSF, cerebrospinal fluid; EBV, Epstein-Barr virus; EEG, electroencephalography; ENAs, extractable nuclear antigens; ERG, electroretinogram; FDG-PET, fluorodeoxyglucose-positron emission tomography; HBV, hepatitis B virus; HCV, hepatitis C virus; IgA/G/M, immunoglobulin A/M/G; MRI, magnetic resonance imaging; MRZ, antibody indices against measles, rubella, and varicella zoster virus; OCT, optical coherence tomography; PCR, polymerase chain reaction; ref., reference; TG, thyroglobulin; TPO, thyroid peroxidase; TSH, thyroid-stimulating hormone; VZV, varicella zoster virus; WBC, white blood cell.*
